# Advances in bladder preservation therapy for muscle-invasive bladder cancer

**DOI:** 10.3389/fonc.2025.1562260

**Published:** 2025-05-01

**Authors:** Ran Zhang, Chang-Xing Ke

**Affiliations:** Department of Urology, The Second Affiliated Hospital of Kunming Medical University, Kunming, China

**Keywords:** muscle-invasive bladder cancer (MIBC), bladder-preserving therapy, comprehensive bladder-preserving therapy, trimodality treatment, multimodality treatment

## Abstract

Bladder cancer is one of the most common genitourinary malignancies. Radical cystectomy (RC) and pelvic lymphadenectomy (PLND) after neoadjuvant chemotherapy have become the accepted gold standard for the treatment of resectable muscular invasive bladder cancer due to its ability to surgically remove tumor tissue as thoroughly as possible and reduce the risk of tumor recurrence and metastasis, thus improving patient survival. However, RC surgery is challenging and associated with many postoperative complications, requiring patients to have good physical condition to tolerate the procedure. Over the years, with the deepening of medical research and the accumulation of clinical practice, the disease spectrum of bladder cancer has changed significantly. At the same time, the treatment modalities for bladder cancer have also been continuously improved and updated, and bladder-preserving treatment programs have gradually emerged and demonstrated reliable efficacy. Bladder-sparing treatment aims to preserve the physiological function of the bladder while controlling tumor growth, thereby improving patients’ quality of life. This approach has led an increasing number of MIBC patients to choose bladder-sparing treatment after considering their individual conditions. In this paper, we review the current methods of bladder-sparing treatment for MIBC patients and related studies to provide a reference for future research.

## 1 Introduction

Bladder cancer (BCa) is one of the most common malignant tumors of the urinary system, ranking 10th among new malignant tumors in the world and 7th among new malignant tumors in men in China, and its incidence rate has been rapidly increasing year by year (1, 2). BCa is more common in men, and the difference in incidence between men and women is about three times. According to the depth of tumor infiltration into the bladder wall, BCa can be classified into MIBC and non-muscle invasive bladder cancer (NMIBC), of which MIBC accounts for about 25% and has a poor clinical prognosis (3). Neoadjuvant chemotherapy followed by RC and pelvic lymph node dissection remains the gold standard for the treatment of resectable MIBC. However, RC is difficult to perform and requires patients to have good cardiopulmonary function to tolerate the surgery; secondly, urinary diversion is required after RC, which often leads to complications such as postoperative infection, intestinal obstruction, intestinal fistula, urinary fistula, lymphocyst, and deep vein thrombosis, which reduces the patients’ quality of life. Studies have shown that nearly 49% of MIBC patients choose bladder preservation therapies (BPT) as a treatment option due to concerns about decreased quality of life after surgery and refusal of urinary diversion (4).

In recent years, changes in the disease spectrum of BCa and the depth of research on BCa treatment have led to the gradual development of comprehensive bladder preservation treatment modalities. There is considerable consensus that bladder preservation therapy can significantly improve the quality of life of some MIBC patients and achieve a long-term prognosis comparable to that of RC, which is a necessary adjuvant treatment for MIBC. There are many BPT treatment options, whether single or comprehensive, that have been shown to be clearly effective in previous studies, but their treatment protocols have not yet reached a unanimous consensus.

## 2 Indications for bladder preservation therapy

Not all patients with MIBC are suitable for bladder-preserving therapy, and some BCa patients with T2 stage on imaging can be considered as an advantageous population after diagnostic electrodesiccation combined with bladder perfusion chemotherapy, with no tumor recurrence during the follow-up period within 3 months. BPT is advantageous for those who meet the following two conditions: ① patient factors: good adherence, good bladder function and capacity; ② tumor factors: T2 stage, single tumor, no combined carcinoma *in situ*, no hydronephrosis, and complete TURBT. Some high-risk patients who are unsuitable or have a high probability of recurrence, the cure rate of forced bladder-preserving treatment will be greatly reduced (5), and may even lead to tumor progression as a result of missing the optimal time for radical surgery. Therefore, it is particularly important to strictly define the indications for BPT. In addition, some genetic monitoring or molecular typing tests can not only clarify the etiology and predict the efficacy of therapeutic drugs for patients, but also their efficacy sensitivity can be used as a screening index for the favorable population, so that more patients with MIBC have the opportunity to preserve their bladder.

## 3 Methods of bladder preservation therapy

At present, the common clinical BPT program includes surgical treatment and non-surgical treatment in terms of treatment means, and includes single treatment program and comprehensive treatment program in terms of treatment content. The contents mainly include: surgical treatment, radiotherapy, chemotherapy and so on. Although it is difficult to achieve satisfactory therapeutic effect with single treatment program, it is an important part of BPT program.

### 3.1 Surgical treatment

The goal of surgical treatment is to remove as much of the visible tumor as possible. Choosing a reasonable surgical method according to the individual differences of patients is the key to BPT. At present, the surgical treatments for BPT tried by scholars at home and abroad mainly include: transurethral resection of bladder tumor (TURBT), holmium laser resection of bladder tumor (HOLRBT),and partial cystectomy (PC).

### 3.2 Chemotherapy

The goal of chemotherapy is to control tumor metastasis and recurrence. Approximately half of MIBC patients have potential metastases prior to surgery. The advantage of chemotherapy is that the drugs can travel through blood vessels throughout the body and effectively control distant metastases. Currently, GC (gemcitabine and cisplatin) regimens have become the first-line chemotherapy regimen, which is attributed to the more obvious advantages of GC regimens in terms of chemotherapeutic efficacy, safety and tolerability, and the studies by Skinner et al. (6) and Stockle et al (7, 8) have demonstrated that adjuvant chemotherapy achieves a more significant benefit in delaying tumor progression. In addition, preoperative chemotherapy may reduce the stage of the tumor, thus making surgical treatment easier (9). A study by Sternberg et al (10) included 104 patients with cT2-T4 bladder cancer undergoing neoadjuvant therapy, some of whom had significantly shorter stages after preoperative neoadjuvant chemotherapy, and 47% of the patients in the study had intact bladders, which opens up the possibility of bladder-preserving therapy for patients with MIBC who have been effectively treated with neoadjuvant chemotherapy.

### 3.3 Radiation therapy

Radiation therapy (RT) is aimed at controlling primary bladder tumors and local lymph nodes. However, as far as current studies are concerned, single radiotherapy cannot achieve the desired clinical outcome, More than two decades ago, a study by Fossa et al. (11) reviewed 263 patients undergoing RC and 271 patients deemed ineligible or unwilling to undergo RC who were converted to high-dose radiation therapy and showed that the 5-year overall survival rate was 48% in the RC group versus 22% in the RT group, and that even with each of the different TNM staging groups, the survival rate for the RC patients was still about twice as high as that for the RT patients. Domestic guidelines also do not recommend routine preoperative radiotherapy for patients with MIBC, because it only leads to a lower tumor stage but does not improve the survival rate of patients, and it is also associated with severe pelvic adhesions after radiotherapy, which further increases the difficulty of performing RC surgery.

### 3.4 Comprehensive treatment with bladder conservation

Current studies have shown that it is difficult to achieve ideal control of tumor recurrence and progression with a single treatment. Currently, trimodality treatment (TMT) or multimodality treatment (MMT), which consists of surgery+chemotherapy+synchronous combined radiotherapy, is the BPT regimen recommended by many national and international consensuses. of BPT regimens. BPT has the advantage of not only achieving comparable therapeutic effects with RC, but also greatly reducing the physical trauma and complications caused by surgery, greatly improving the postoperative outcomes of patients, and reducing the number of complications. The advantage of BPT is that it cannot only achieve the same therapeutic effect as RC, but also greatly reduce the physical trauma caused by surgery, reduce the complications, and greatly improve the quality of life of patients after surgery, therefore, domestic and foreign urological guidelines recommend TMT or MMT as another recommended treatment option for MIBC patients in addition to RC.

#### 3.4.1 TMT triple therapy

The TMT regimen has demonstrated consistent efficacy in numerous large-scale studies conducted domestically and internationally. Giacalone et al. (12) performed TURBT in combination with radiotherapy in 475 patients with MIBC in stages T2 to T4 from 1986-2013. The disease specific survival (DSS) rates at 5, 10, and 15 years of follow-up were 66%, 59%, and 56%, respectively, and the corresponding OS was 57%, 39%, and 25%, respectively. With the continuous development of surgical techniques and the optimization of simultaneous radiotherapy regimens, the CR of the BPT combination regimen increased from 66% in the early stage (1986-1995) to 88% in the recent stage (2005-2013), and the 5-year OS increased from 53% in the early stage to 75% in the recent stage, and meanwhile, the 5-year DSS also increased from 60% to 84%, while the risk of remedial RC decreased from 42% to 16%. The results of this study provide confidence for more patients who seek to preserve their bladder, as well as hope for patients with contraindications to surgery. In order to enhance the effect of local tumor control and to reduce the chance of pelvic lymph node metastasis as well as distant circulating micrometastasis, radiotherapy is usually adjuvanted after cTURBT, and it is standard practice to irradiate both the bladder cancer tumor as well as the pelvic lymph nodes in the radiotherapy regimen. However, the adverse effects of radiotherapy necessitate caution. The International Urological Association has recommended limiting the irradiation dose to 65 Gy for the bladder and 50 Gy for the pelvis (13). Most domestic and international guidelines underscore the necessity of meticulous patient evaluation and precise screening to ascertain eligibility for TMT regimens. In order to optimize the efficacy of BPT, the 2022 NCCN guidelines recommend that patients suitable for TMT meet specific criteria, including the presence of a solitary tumor in the bladder, with a maximum diameter < 6 cm, the absence of tumor-induced hydronephrosis, the absence of extensive or multifocal CIS, and the presence of adequate bladder function (14). The indications and contraindications for TMT are outlined in **Table 1** (5, 15–17)

**Table 1:** Presents a comprehensive list of indications and contraindications for bladder preservation therapy.

Absolute indications	Relative indications	Contraindications
T2	T3a, very high riskT1G3	T3b-T4b
No hydronephrosis	Bladder dysfunction	Tumor-associated hydronephrosis
No carcinoma in situ	Sporadic carcinoma in situ	Lymph node positivity
TURBT with complete resection of visible tumor	Incomplete TURBT	History of pelvic radiotherapy
Single lesion	Multiple lesions	Prostate invasion
Good bladder function		Intolerance of chemotherapy

Comprehensive treatment based on partial cystectomy is defined as TMT supplemented by PC and PLND, which is indicated for patients with pathological type of uroepithelial carcinoma, tumor confined to less than one-fourth of the total bladder surface area, tumor not invading the neck of the bladder, absence of extensive CIS, and absence of distant metastases. Koga et al. (18) implemented a PC-based combination therapy model in 187 MIBC patients with cT2-T4, and the results showed that all the patients who received the combination therapy did not suffer from MIBC recurrence. The results of this study suggest that our combined PC can improve local tumor control by surgical removal of residual tumors after CRT and BPT. Kijima and his team reported a retrospective study in which 154 Japanese patients with MIBC underwent comprehensive PC-based surgery, which not only resulted in good preservation of bladder function, but also in excellent tumor control, with a 5-year OS of 91% and a local recurrence rate of only 4% (15). In a study comparing RC surgery and PC surgery, Zhang et al. (19) found that partial cystectomy with bladder perfusion therapy achieved comparable tumor control to radical cystectomy in patients with limited isolated T2N0M0 and T3N0M0 muscularly invasive bladder cancer. However, patients in the PC group had significant advantages in terms of operative time, intraoperative bleeding, intraoperative and postoperative blood transfusion, preoperative preparation time, total hospitalization time, postoperative recovery time, operative cost, and operative complications. While this modality can further control tumor recurrence, it also increases the economic burden of patients with the risk of postoperative complications. Consequently, most patients who intend to preserve their bladders still express concern. However, it remains a desirable option for patients with solitary tumors in the bladder and for those who choose to have tumor recurrence after BPT.

#### 3.4.2 TMT or MMT combined with immunosuppressive therapy

Immunosuppressant therapy is the application of antibodies against programmed death receptor 1 and its ligand (programmed death-1/programmed death-ligand1, PD-1/PD-L1), which kills cancer cells using the body’s own immune system by blocking the PD-1/PD-L1 signaling pathway. Immune checkpoint inhibitors, exemplified by PD-1/PD-L1 monoclonal antibodies, have demonstrated significant potential in enhancing the efficacy of advanced uroepithelial cancer treatment. Immunotherapy drugs primarily encompass immune checkpoint inhibitors (ICIs), including anti-PD-1, PD-L1, and CTLA-4 antibodies. A total of five ICIs, including Atilizumab, Navulizumab, and Pembrolizumab, have received approval in the U.S. for the treatment of various stages of bladder cancer (20). In recent years, ICIs drugs have been widely used in the treatment of bladder cancer, and their efficacy has been widely recognized. At present, the U.S. Food and Drug Administration has approved six ICIs for clinical application in bladder cancer, including two PD-1 inhibitors (Navulizumab and Pembrolizumab), three PD-L1 inhibitors (Atalizumab, Durvalumab, and Avelumab), and one CTLA-4 inhibitor (Ipilimumab). The challenge of eradicating intermediate- and late-stage bladder cancer, which has a high recurrence rate, remains unresolved. The advent of immunotherapy has engendered optimism among patients afflicted with middle- and late-stage bladder cancer. A notable proportion of patients have exhibited substantial efficacy with immunotherapy in the neoadjuvant stage, suggesting a potential for enhanced efficacy when combining immunotherapy with bladder preservation therapy, thereby achieving a higher success rate in bladder preservation. A notable concern is the toxicity of chemotherapy, which can lead to BPT failure, particularly in elderly patients. The low toxicity and synergistic anti-tumor effects of immunotherapy can potentially compensate for this challenge. It is hypothesized that the incorporation of immunotherapy into bladder preservation therapy will enhance its efficacy. Weickhardt et al. (21) included in a single-arm phase II trial of Pembrolizumab in combination with radiotherapy in 28 patients with MIBC in cT2-T4aN0M0, which showed a complete remission rate of 88% and low drug toxicity. and Balar et al. (22) included 370 patients with locally advanced and unresectable or metastatic uroepithelial carcinoma unsuitable for cisplatin treatment who received pembrolizumab, and the results confirmed antitumor activity and acceptable tolerability in patients with cisplatin-ineligible uroepithelial carcinoma, the majority of whom were elderly, had poorer prognostic factors, or had serious comorbidities. In light of this result, paborizumab has emerged as a new treatment option for patients ineligible for cisplatin or unsuitable for chemotherapy. Toripalimab, the first domestically produced PD-1 inhibitor, was approved by the State Drug Administration of China for the treatment of uroepithelial carcinoma in April 2021 due to its reliable safety and excellent efficacy. A study by Sheng et al. (23) included 151 patients with metastatic uroepithelial cancer, and the results demonstrated the better safety, efficacy, and also confirmed that PD-L1 expression can be used as a good predictive marker for Toripalimab. It is anticipated that the future will bring an increase in the availability of immunosuppressant medications for the treatment of muscle-invasive bladder cancer, along with the development of updated bladder-preserving treatment regimens to guide the selection of future BPT regimens.

#### 3.4.3 TMT or MMT combined with targeted therapy

Anti-angiogenic drugs, fibroblast growth factor receptor inhibitors, human epidermal growth factor family receptor inhibitors, and MET pathway inhibitors are currently used in targeted therapy of bladder cancer. Fibroblast growth factor receptor (FGFR) has four members, namely FGFR1-4. FGFR can regulate vascular growth together with vascular endothelial growth factor and platelet-derived growth factor receptor, FGFR3 mutation has the highest frequency of mutation in bladder cancer genes, and is considered to be one of the most promising molecular One of the predictive markers, Erdafitinib is an oral small molecule inhibitor targeted to patients with bladder cancer who have a family of mutations in the FGFR gene. Erdafitinib was approved in the U.S. in January 2024 for the treatment of locally advanced or metastatic urothelial cancer with susceptible FGFR3 gene alterations. Matsubara et al. (24) demonstrated in detail and comprehensively the remarkable efficacy and overall safety of Erdafitinib in clinical applications. Another study by Siefker-Radtke et al. (25) included 101 patients with metastatic uroepithelial carcinoma with FGFR mutations treated with Erdafitinib, and the results of the study similarly confirmed the reliable efficacy and safety of Erdafitinib. From these research results, it is easy to see that the emergence and application of new targeted drugs such as Erdafitinib have brought a new direction of thinking in the field of cancer treatment. In addition, several studies of antibody-drug conjugates (ADCs) combined with immunotherapy are currently underway, which not only confirm the excellent efficacy of ADC drugs in the treatment process, but also reveal the possible synergistic promotion between ADC and immunotherapy from the side (26, 27). Three ADC drugs have been approved for the treatment of urothelial carcinoma: Enfortumab (nectin-4 antibody-MMAE coupling), Disitamab Vedotin (HER2 antibody-MMAE coupling) and Gosartuzumab (Trop-2 antibody-SN-38 coupling), of which Enantiomab and Gosatuzumab are approved in the U.S., and Disitamab Vedotin(RC48) is approved in China. Sheng et al. included 43 patients with chemotherapy-failed locally advanced or uroepithelial cancers with strong positive HER2 expression and showed that RC48- ADC has good efficacy and a manageable safety profile in patients with locally advanced or uroepithelial cancers with strong positive HER2 expression who have failed at least one line of systemic chemotherapy. However, large multicenter randomized controlled trials of TMT or MMT in combination with targeted therapeutic agents for MIBC patients are currently in a gap phase in China. We have enough reasons to believe that with the continuous deepening and development of medical research, more precise targeted drugs will emerge in the field of postoperative adjuvant therapy for bladder cancer in the future. The emergence of these drugs will not only provide a richer and more solid basis for bladder cancer patients to individually choose postoperative adjuvant therapy, but will also most likely open a new starting point for targeted precision therapy of bladder cancer and open a new chapter in bladder cancer treatment.

## 4 Analysis of recurrence rates and long-term toxicity after comprehensive bladder-preserving therapy

Although bladder-preserving combination therapy is more effective in terms of tumor control, recurrence rates and long-term toxicity are part of the equation to be considered. One study (12) reported long-term results of bladder-preserving therapy using triple therapy (radiation, chemotherapy, and transurethral cystectomy for bladder tumors) at Massachusetts General Hospital. The study showed a 5-year overall survival rate of 57%, a local recurrence rate of 31%, and a distant metastasis rate of 31%. Fong et al. (28) evaluated the efficacy of TMT in muscle-invasive bladder cancer. The results showed a 5-year overall survival rate of 50%-60% and a local recurrence rate of 20%-30%. For patients with local tumor recurrence after comprehensive treatment, RC surgery is still recommended, while immunotherapy and targeted therapy can be new options for patients who develop distant metastases. Adverse reactions and long-term toxicities of bladder-preserving combination therapy are most commonly seen with radiotherapy and chemotherapy. Long-term adverse and toxic reactions to bladder-preserving combination therapy are most common in the genitourinary and gastrointestinal tracts., a study by Efstathiou et al (29) included 285 patients with MIBC undergoing bladder preservation therapy and found that common grade 3-5 adverse events were hematologic, gastrointestinal, and genitourinary toxicities, but thankfully, the incidence of late toxicity with bladder preservation therapy was low and the occurrence of long-term adverse events and toxicity was mostly considered to be related to the choice of treatment regimen. The treatment of adverse and toxic reactions is still a problem waiting to be solved, and the current means of treatment is only symptomatic treatment according to different reactions, and if the reaction is serious, the drug can be discontinued according to the actual situation. Fortunately, immune drugs and targeted drugs have better control of adverse reactions and toxic reactions, which brings new treatment options for patients who are intolerant to radiotherapy and chemotherapy.

## 5 Long-term follow-up of BPT

Follow-up of patients with muscle invasive bladder cancer with bladder preservation mainly consists of cystoscopy and imaging, and urine cytology and urine tumor markers are also valuable. cystoscopy every 3 months for 2 years, every 6 months starting in the 3rd year, and annually after 5 years. In addition, CT or MRI of the chest and abdomen every 3 to 6 months for 2 years, and then annually thereafter, and PET-CT if metastasis is suspected (30). Here I list a schedule of follow-up visits organized by Chinese experts that have gained consensus, as detailed in **Table 2**.

**Table 2:** Follow-up schedule after bladder preservation therapy in MIBC patients.

Inspection items and frequency of inspection	First year	Second year	Third year	Fourth year	Fifth year	Years 6-10	After 10 years
Cystoscopy	1 time every 3 months	1 time every 3 months	1 time every 6 months	1time every 6 months	1 time every 1 year	1 time every 1 year	When clinically suspicious symptoms appear
Imaging Examination	1. CTU or MRU every 3–6 months; 2. Chest X-ray or chest CT every 3–6 months; 3. Systemic FDG PET-CT if metastasis suspected	1. CTU or MRU every 3–6 months; 2. Chest X-ray or chest CT every 3–6 months; 3. Systemic FDG PET-CT if metastasis suspected	1. 1 CTU or MRU per year; 2. 1 chest X-ray or chest CT per year; 3. Systemic FDG PET-CT when metastasis is suspected	1. 1 CTU or MRU per year; 2. 1 chest X-ray or chest CT per year; 3. Systemic FDG PET-CT when metastasis is suspected	1. 1 CTU or MRU per year; 2. 1 chest X-ray or chest CT per year; 3. Systemic FDG PET-CT when metastasis is suspected	Imaging based on clinical need	Imaging based on clinical need
Blood Tests	Blood liver function, kidney function, electrolytes, routine blood tests every 3–6 months	Perform blood liver function, kidney function, electrolytes and routine blood tests as clinically indicated	Perform blood liver function, kidney function, electrolytes and routine blood tests as clinically indicated	Perform blood liver function, kidney function, electrolytes and routine blood tests as clinically indicated	Perform blood liver function, kidney function, electrolytes and routine blood tests as clinically indicated	Perform blood liver function, kidney function, electrolytes and routine blood tests as clinically indicated	Perform blood liver function, kidney function, electrolytes and routine blood tests as clinically indicated
Urine Tests	Urine cytology every 6–12 months	Urine cytology every 6–12 months	Urine cytology as clinically indicated	Urine cytology as clinically indicated	Urine cytology as clinically indicated	Urine cytology as clinically indicated	Urine cytology as clinically indicated

## 6 Future perspectives for bladder-preserving therapy

In 2018, Sjödahl’s study (31) used a combination of tissue microarray (TMA) and immunohistochemistry (IHC) techniques to classify bladder cancer into molecular subtypes using specific protein markers. The classification process, which included staining, evaluation, data adjustment and validation, analyze the subtype classification of urothelial carcinoma at different pathologic stages and concluded five subtypes, namely: uroepithelial-like, genomically unstable, basal/SCC-like, mesenchymal-like, and small-cell/neuroendocrine (See **Table 3** for specific categorization), Tumor molecular subtype stratification is key to cancer research, and mRNA or protein analysis is a commonly used tool. Compared to mRNA expression profiling, IHC technology based on tissue *in situ* analysis can complement the classification information at the tumor cell phenotype level. In this study, Sjödahl combined tissue microarray (TMA) and IHC techniques to classify the molecular subtypes of uroepithelial carcinomas from Ta to ≥T2 stages, and the method is generalizable and has been validated in multiple cohorts, although there is still room for improvement. in 2020. Kamoun et al (32) arrived at a consensus classification defining the following six subtypes: luminal papillary (24%), luminal nonspecified (8%), luminal unstable (15%), stroma-rich (15%), basal/squamous (35%), and neuroendocrine-like (3%). The proposal and development of molecular typing provides new directions and guidance for the management of MIBC, by which we can not only understand the pathogenesis of MIBC, a cancer with high heterogeneity, but also predict reliable treatment modalities and prognosis. However, most of the published articles are retrospective studies, and there is a lack of large-sample, multicenter prospective studies and reports, so whether the appealing treatment method is accurate and effective still deserves further discussion and research.

**Table 3:** Markers used to define the phenotypes of muscle invasive bladder cancer.

Uro	FGFR3 +	CCND1 +	RB1 +	CDKN2A (p16) -
GU	FGFR3 -	CCND1 -	RB1 -	CDKN2A (p16) +
Basal/SCC - like	KRT5 +	KRT14 +	FOXA1 -	GATA3 -
Mes - like	VIM +	ZEB2 +	CDH1 -	EPCAM -
Sc/NE - like	TUBB2B +	EPCAM +	CDH1 -	GATA3 -

Although some progress has been made in BPT regimens, there is still a need for further improvement. First, to address the problem of tumor recurrence after extensive treatment, is there a more appropriate protocol to avoid further tumor progression, and how to adjust the toxic and side effects of chemotherapy and radiotherapy to achieve a balance between therapeutic efficacy and appropriateness. In addition, more reliable studies are needed to confirm the efficacy of targeted immunotherapy. Finally, there is still an urgent need to find suitable biomarkers that can be used as clinical predictors to provide clinicians with more accurate references to make more precise clinical treatment decisions.

The goal of bladder-preserving therapy is to improve patients’ quality of life under the premise of maximally controlling tumor progression, and due to the existence of individual differences among patients, the choice of treatment protocols is inevitably individualized. In the future, the process of bladder-preserving therapy should be more rigorous, and patients and their families should be fully and carefully informed about the benefits and potential risks of BPT therapy, including possible side effects during treatment and the possibility of recurrence after treatment. At the same time, the patient’s personal wishes, family financial situation, physical tolerance level, and other factors should be fully considered and the pros and cons weighed before making a prudent decision. In addition, good patient compliance is a critical factor and regular long-term follow-up is essential. Only through long-term follow-up can potential problems be detected in time and the treatment program adjusted. Based on these points, it is possible for most MIBC patients to receive individualized and optimal treatment.

## 7 Optimizing patient choice is key to bladder preservation

Bladder preservation therapy has extremely strict criteria for patient selection, but this process is often the most difficult because it involves not only a comprehensive analysis and evaluation of the patient’s condition and relevant examination data, but also a combination of the physician’s relevant treatment experience. Having an accurate predictive tool can solve this problem. Zhang et al. (33) retrospectively studied the CTU characteristics of 441 MIBC patients and concluded that the CT-based imaging histology prediction model for preoperative assessment of muscle infiltration in bladder cancer has good diagnostic effect, which is expected to provide a reference for personalized treatment and prognosis judgment of bladder cancer. However, the study has limitations such as small sample size and selective bias, and the accuracy of the model needs to be improved, and a more comprehensive prediction model can be constructed by combining more information in the future. It is worth noting that with the development of the times, Artificial Intelligence (AI) is gradually applied to all aspects of our lives, and in the healthcare field as well. In China, some hospitals have already introduced AI to do non-decision-making tasks, such as cleaning and some basic paperwork, but AI has a huge potential for medical decision-making. The study by Ma et al. (34) proposes the application of AI in the diagnosis and treatment of bladder cancer, and the breakdown can be divided into the application of AI in the diagnosis, treatment, and prediction of prognosis of bladder cancer. In the diagnostic process, such as cystoscopy, AI can improve diagnostic accuracy and assist in categorization; in CT, MRI, and ultrasound, it can improve the image analysis ability; and in urinary cytology, it can enhance the sensitivity of the test; In molecular biology, it can help analyze molecular markers. In addition, AI can provide more reliable results. Application of AI in bladder cancer treatment: it plays a role in all aspects of radiation therapy. It can reconstruct low-dose CT images, perform bladder segmentation and target area outlining, assist in treatment planning, and carry out quality assurance work, which is promising although it faces the problems of model standardization, etc. AI can also predict patients’ survival rate, recurrence rate, and response to treatment, as well as carry out pathological assessment, analyze genetics and molecular biology related indexes, and provide a basis for prognostic judgment. However, AI decision-making requires the incorporation of a large amount of data, raising numerous privacy, ethical issues and clinical acceptance that remain significant challenges. Although there are still many challenges in predicting the prognosis of patients with MIBC, it is believed that in the future there will be more accurate means of screening patients suitable for bladder-preserving therapy.

## 8 Conclusion

Comprehensive treatment regimens with bladder preservation have shown impressive results, with efficacy comparable to radical cystectomy (RC). In particular, TMT is not inferior to conventional RC in terms of tumor control (12). However, comprehensive bladder-preserving treatment involves a variety of therapeutic tools, and there are individual differences in treatment efficacy; in addition, treatment decisions made by physicians with professional knowledge and clinical experience are directly related to the direction and quality of the treatment plan; the patient’s own willingness to play a decisive role and attitude of active cooperation with the treatment often leads to a better therapeutic effect; and the patient’s energy and financial status should not be neglected. The long-term treatment process requires patients to invest a lot of time and financial costs, if patients have difficulties in these aspects, it may affect the continuity and integrity of the treatment; in addition, the level of medical facilities available to the patient, such as advanced examination equipment, high-quality drug resources, etc., will also have a decisive impact on the therapeutic effect. Therefore, physicians must fully and carefully inform patients and their families about the benefits and potential risks of BPT treatment, including possible side effects during treatment and the possibility of recurrence after treatment. At the same time, the patient’s personal wishes, family financial situation, physical tolerance level, and other factors should be fully considered and the pros and cons weighed before making a prudent decision. In conclusion, BPT provides new ideas for MIBC patients who are intolerant to RC and unwilling to undergo RC, but the establishment of a standardized diagnostic and therapeutic process is still a goal that academics are striving for, and the addition of immunosuppressive therapy and targeted therapy greatly enriches the BPT model. It is believed that in the near future, BPT will become the first choice for more and more MIBC patients.
